# Impact of the COVID-19 Pandemic on Urologic Oncology Surgery: Implications for Moving Forward

**DOI:** 10.3390/jcm11010171

**Published:** 2021-12-29

**Authors:** Rossella Guerrieri, Lucrezia Rovati, Paolo Dell’Oglio, Antonio Galfano, Luca Ragazzoni, Paolo Aseni

**Affiliations:** 1 Emergency Department, ASST Grande Ospedale Metropolitano Niguarda, 20162 Milano, Italy; rossella.guerrieri@ospedaleniguarda.it (R.G.); paoloaseni@gmail.com (P.A.); 2 School of Medicine and Surgery, University of Milano-Bicocca, 20126 Milano, Italy; 3 Department of Urology, ASST Grande Ospedale Metropolitano Niguarda, 20162 Milano, Italy; paolo.delloglio@ospedaleniguarda.it (P.D.); antonio.galfano@ospedaleniguarda.it (A.G.); 4 CRIMEDIM—Center for Research and Training in Disaster Medicine, Humanitarian Aid and Global Health, Università del Piemonte Orientale, 28100 Novara, Italy; luca.ragazzoni@med.uniupo.it; 5 Department of Biomedical and Clinical Sciences “L. Sacco”, Università degli Studi di Milano, 20157 Milano, Italy

**Keywords:** COVID-19, pandemic, SARS-CoV-2, urologic oncology surgery, prostate cancer, kidney cancer, bladder cancer

## Abstract

The COVID-19 pandemic has caused the destruction of routine hospital services globally, leading to an increase in the backlog of elective surgery cases. The aim of the study was to retrospectively investigate the pandemic’s impact on the urologic oncology surgical activity of a high-volume center located in Milan, Italy. The number and type of procedures performed in 2020 during the COVID-19 pandemic was evaluated using 2019 data as control. Waiting times for each surgical procedure were compared, on a bimonthly basis, between the two different years. Overall, a 26.7% reduction in the number of urologic oncology surgeries between 2019 and 2020 was observed (2019: 720, 2020: 528). Both the main indication for surgery and the type of procedure performed significantly differed between 2019 and 2020 (all *p* < 0.0001), with a decrease in the number of radical prostatectomies and an increase in the number of radical cystectomies and radical nephrectomies/nephroureterectomies performed in 2020. Waiting time decreased by 20% between 2019 and 2020, with the most significant reduction seen after the first wave of the COVID-19 pandemic (July–October 2020), in particular for partial nephrectomy and radical prostatectomy, possibly due to the underdiagnosis of cases. In conclusion, in accordance with recommendations by international urological societies on prioritization strategies for oncological procedures, a higher proportion of surgeries for high-risk tumors was performed in 2020 at our center at the expense of procedures for lower risk diseases; however, future implications for patients’ prognosis still need to be determined.

## 1. Introduction

The COVID-19 pandemic has interrupted routine hospital services globally. A vast number of surgical operations have been cancelled or postponed owing to the disruption caused by COVID-19 [1]. Delays in treatment, especially for time-sensitive surgical diseases, such as malignancy, may result in increased disease severity, morbidity and mortality [2,3]. Governments should therefore mitigate against this significant burden on patients by developing recovery plans and implementing strategies to restore routine diagnostic and surgical activity safely [4,5].

Lombardy has been the first and the most severely affected region of Italy during the first and second waves of the COVID-19 pandemic in 2020. Local and national authorities recommended drastically reducing surgical case volumes by cancelling elective procedures and redirecting both personnel and equipment resources for anticipated COVID-19 patients [6]. Our hospital, a high-volume hospital located in Milan, Italy, mandated cancellation of all elective procedures both during the first and the second COVID-19 wave, in March and April 2020 and again in November and December 2020.

The changes in the management of urologic activities caused by COVID-19 have already been reported by some authors [7,8,9,10,11]. In particular, the urology team of our institution has described a reduction in all urologic activities performed during the first COVID-19 wave in the three largest public hospitals in Lombardy (Brescia, Bergamo and Milan) [12]. In accordance with recommendations by international urological societies, some urologic oncology surgical procedures have undergone a significant reduction in volume based on specific prioritization strategies, leading to an increased backlog of cases and possibly a worse prognosis for some patients [13,14,15,16,17,18,19,20,21,22,23].

This study aims to analyze the impact of the COVID-19 pandemic on the urologic oncology surgical activity of our institution, evaluating the number and type of procedures performed in 2020 during the COVID-19 pandemic using 2019 data as control. Moreover, this study analyzes the difference in waiting time for each surgical procedure between the two different years.

## 2. Materials and Methods

### 2.1. Study Design, Study Population and Outcome Measures

A retrospective study was performed on all adult patients (age ≥ 16 years) who underwent urologic oncology surgery at Niguarda Hospital, a high-volume center located in Milan, Italy, between 2019 and 2020. The study also included a smaller group of patients that in 2020 were initially assessed and followed-up at Niguarda Hospital although, due to the COVID-19 pandemic, they were subsequently operated on by the Niguarda urology surgery team at a different institution, the European Institute of Oncology (IEO), a comprehensive cancer center located in Milan, Italy.

Data were collected for all adult patients treated with partial nephrectomy, radical nephrectomy or nephroureterectomy, transurethral resection of bladder tumor (TURBT), radical cystectomy and radical prostatectomy for an oncological indication. Patients included in the study underwent either elective or emergency hospital admission. The data were collected retrospectively starting from January 2019 through December 2020. Data included for each patient comprised: sex; age at surgery; nationality; type of hospital admission; length of hospitalization; primary diagnosis; type of surgery; date of surgery; date in which surgical indication was given.

The primary outcome was the difference in waiting time, defined as the interval between the date of the visit during which surgical indication was given and the date of surgery, between the COVID-19 pandemic months of 2020 and the corresponding months of 2019. The months of January and February were included in the study, even if the COVID-19 pandemic in Italy was not yet fully blown, to have an internal control.

The study was conducted in accordance with the Declaration of Helsinki and the protocol was approved by the Ethics Committee of Niguarda Hospital.

### 2.2. Statistical Analysis

We calculated the monthly urologic oncology surgical volumes for 2019 and 2020, comparing each month of 2020, which comprised periods affected by the COVID-19 pandemic, with the same month of 2019. Waiting time for each surgical procedure was compared, on a bimonthly basis, between the two different years.

All analyses were conducted using Stata software version 17.0 (StataCorp, College Station, TX, USA). Quantitative data are expressed as mean ± standard deviation or median (interquartile range), as needed. Categorical data are expressed as numbers (percentage). Comparisons between two categorial variables were performed using Pearson’s chi-squared test or Fisher’s exact test. Comparisons between two groups of continuous data were performed using unpaired t-test or Mann–Whitney test. *p*-values < 0.05 were considered statistically significant.

## 3. Results

### 3.1. Study Cohort

A total of 1248 patients were included in the study. In particular, 720 patients operated on in 2019 and 528 patients operated on in 2020 were assessed in this work. Of the 528 procedures performed in 2020, 100 (18.9%) were performed at the European Institute of Oncology (IEO) by the Niguarda Hospital urology surgery team. Patients’ characteristics are summarized in Table 1.

The majority of patients were male, even if there was a slight increase in the proportion of female patients operated on in 2020 compared to 2019. Both the average age of the patients and the proportion of foreign patients who underwent urologic oncology surgery remained stable between 2019 and 2020 (Table 1).

Most of the surgical procedures were performed after elective hospital admission; 4% and 6% of patients needed emergency hospital admission in 2019 and 2020, respectively. The absolute number of emergency hospital admissions remained roughly constant between the two years (31 in 2019 and 34 in 2020). No difference was observed in the average length of hospital stay between the two years (Table 1).

Both the main indication for surgery and the type of procedure performed differed significantly between 2019 and 2020 (*p*-value < 0.0001 for both comparisons). In particular, in 2020 there was a significant decrease in the number of radical prostatectomies performed for prostate cancer (from 264 in 2019 to 125 in 2020). In addition, despite a decrease in the total number of surgeries, an increase in the number of radical cystectomy (from 32 in 2019 to 37 in 2020) and radical nephrectomy/nephroureterectomy (from 31 in 2019 to 46 in 2020) performed for bladder cancer and kidney or upper tract urothelial carcinoma (UTUC), respectively, was observed in 2020 compared to 2019.

### 3.2. Number of Urologic Oncology Surgeries Performed

**Figure 1:**
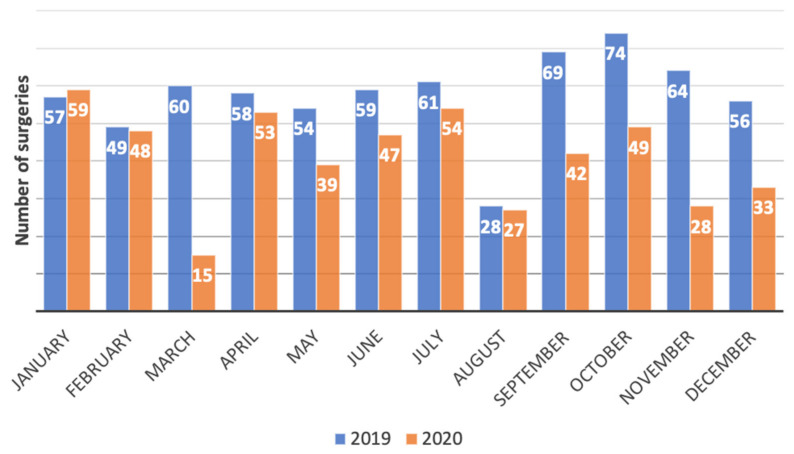
Monthly distribution of the overall number of urologic oncology surgeries performed by the Niguarda urology surgery team: comparison between 2019 and 2020.

The total number of urologic oncology surgeries performed by the Niguarda urology surgery team underwent a 26.7% reduction in volume between 2019 and 2020 (from 720 procedures in 2019 to 528 procedures in 2020). The monthly trend in urologic oncology surgeries is shown in Figure 1.

The greater reduction in the number of surgeries was seen in March 2020, at the beginning of the COVID-19 pandemic: in fact, surgical volume decreased from 60 to 15 procedures compared with March 2019 (75% decrease). After the beginning of the COVID-19 pandemic, surgical volume remained lower throughout the whole of 2020. However, the second most important reduction was seen in November 2020, at the beginning of the second wave of the COVID-19 pandemic in Italy (56% reduction, from 64 to 28 surgeries).

No difference was seen, as expected, in the number of surgeries performed in the months of January and February, before the beginning of the restrictions imposed by the COVID-19 pandemic at our institution.

### 3.3. Waiting Time

**Figure 2:**
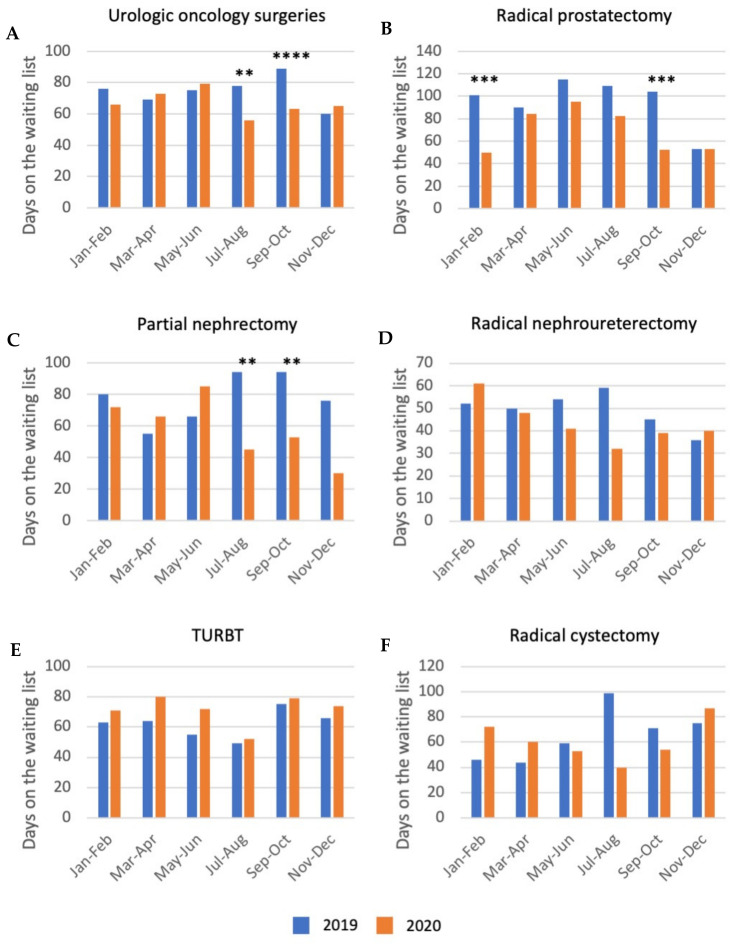
Bimonthly distribution of the waiting time for (**A**) the overall number of urologic oncology surgeries, (**B**) radical prostatectomy, (**C**) partial nephrectomy, (**D**) radical nephroureterectomy (includes also radical nephrectomy), (**E**) transurethral resection of bladder tumor (TURBT) and (**F**) radical cystectomy: comparison between 2019 and 2020. Legend: ** *p*-value ≤ 0.01; *** *p*-value ≤ 0.001; **** *p*-value ≤ 0.0001.

Waiting time for surgery decreased by 20% between 2019 and 2020, from an average of 72 days (IQR 33-111 days) to an average of 58 days (IQR 35–90 days). The decrease in waiting time was seen mainly for patients operated on during the months following the first wave of the COVID-19 pandemic, from July to October 2020 (Figure 2). Indeed, waiting time was significantly lower for patients operated on in July–August and September–October 2020 compared to the same bimesters of 2019 (*p*-value = 0.0046 and *p*-value < 0.0001, respectively). In particular, a significant decrease in waiting time was seen in those months for partial nephrectomy and radical prostatectomy (Figure 2). A significant decrease in waiting time was also seen only for prostate cancer, in the months of January and February 2020.

## 4. Discussion

After the beginning of the COVID-19 pandemic in Italy in February 2020, all elective non-urgent surgical activities were interrupted in order to provide resources for the emerging epidemic and in fear of exposing patients to in-hospital SARS-CoV-2 infection [1,4,5,6]. Niguarda Hospital, one of the largest public hospitals in the Lombardy region of Italy, was designated as a COVID-19 hub by the regional government and focused mainly on the treatment of COVID-19 patients. However, being a high-volume oncologic referral center, the regulatory authorities allowed the urology surgery team to keep performing major cancer surgery at Niguarda Hospital and, in part, in another medical structure, the European Institute of Oncology (IEO) [12]. However, surgical and postoperative care were undertaken by the same staff at both hospitals, reducing differences in patients’ management. The elective surgical activity was interrupted both during the first and the second COVID-19 wave, in March and April 2020 and again in November and December 2020 to face logistic pandemic challenges. From May to October 2020, elective surgical activity was gradually started again.

The main finding of our study is that, despite a significant decrease in the number of urologic oncology surgeries performed during the months of the COVID-19 pandemic, the average waiting time from diagnosis to surgery decreased in 2020 when compared to 2019. The decrease in waiting time was seen mainly after two months from the end of the first wave, from July to October 2020, before the beginning of the second wave. A significant reduction in waiting time in those months was seen for partial nephrectomy and radical prostatectomy; a reduction trend was seen also for radical nephrectomy/nephroureterectomy and radical cystectomy. A decrease in elective oncological surgical activity has already been reported by other studies, also in the urologic oncology field; however, this finding has been associated to an increase in the average waiting time for surgery [3,24,25,26,27,28,29,30]. Our findings might be explained by the fact that, during the months of the COVID-19 pandemic, fewer cancer diagnoses were performed at our center, leading to a shorter patients’ waiting list for surgery. In particular, the decrease in the rate of prostatic biopsies (5% of the usual volume), cystoscopies (20% of the usual volume) and urological consultations (15% of the usual volume) performed during the COVID-19 pandemic could have led to an underdiagnosis of cases of asymptomatic and localized cancers [12]. Indeed, recent studies are highlighting the relevance of the decrease in screening procedures during the COVID-19 pandemic, which might lead to increased cancer mortality [31,32,33,34].

Another important finding of our study is the difference in the type of urologic oncology surgical procedures performed in 2020 compared to 2019. Indeed, an increase in surgeries performed for high-risk tumors, namely radical nephrectomy/nephroureterectomy and radical cystectomy, and a decrease in surgeries performed for lower-risk tumors, namely partial nephrectomy, TURBT and radical prostatectomy, were observed in this study. This is in accordance with prioritization strategies recommended by different international urological societies, which suggest the avoidance of delays in performing radical nephrectomy for advanced kidney tumor, radical nephroureterectomy for high-grade UTUC and radical cystectomy for high-risk muscle-invasive bladder cancer [13,14,15,16,18,19,20,21,28,35,36]. On the other hand, other urologic oncology surgical procedures, including radical prostatectomy for low-to-intermediate-risk prostate cancer, might be postponed for a certain period of time [36,37,38].

Interestingly, the decrease in the number of surgeries performed at our center did not lead to an increase in the number of emergency hospital admissions for urologic tumors, in accordance with the current literature [7,9]. This could mean that delayed surgery does not cause an increase in the need for emergency room visits and emergency surgeries that could further stress the healthcare system during the pandemic.

However, several consequences might arise from the observed decrease in the number of urologic oncology surgical and diagnostic procedures performed during the COVID-19 pandemic. Recent research works demonstrated how a decrease in diagnosis and a delay in surgical treatment of early tumors might lead to cancer upstaging and worse patients’ prognosis [14,22,23,33,34,39]. Moreover, the reduction in surgeries for localized disease might lead to subsequent higher surgical complexity, with greater risk of complications, and lower possibility of training for residents and young surgeons [40,41]. Further studies are needed to compare tumor stages before and after COVID-19 pandemic, to better ascertain which diagnostic and therapeutic interventions can be safely postponed without leading to an increase in the proportion of advanced cancers.

In this context, different solutions can be implemented to decrease the burden of delayed patient diagnosis, treatment and follow-up. One option is to assess and treat oncologic patients in COVID-free specialized tertiary structures, such as the European Institute of Oncology (IEO) in Milan. In addition, telemedicine services can be implemented, in particular for follow-up visits that more frequently do not require physical examination and instead require only an assessment of previously performed blood and imaging tests. This would leave free slots for in presence first visits and therefore contribute to the optimization of limited resources [42,43,44,45,46].

The findings of our research must be interpreted carefully due to several limitations of this study. First, this is a monocentric study performed in a large public Italian hospital that was heavily affected by the COVID-19 pandemic. Different findings might be observed in other regions of Italy or in other countries, especially when also taking into account private institutions. Moreover, some of the results could have been impacted by the fact that some patients were operated on in another institution (IEO), even if this was performed by the same surgical team, during 2020. Regarding prostate cancer, the decrease in the waiting time for radical prostatectomy seen in the first months of 2020, before the beginning of the COVID-19 pandemic, might be related to a slight increase in the number of prostate surgeries performed at the end of 2019 and must be considered when evaluating these results. Further studies on a larger number of patients from different institutions correlating the changes in surgical activity with the number of novel urologic tumor diagnosis and the stages of treated tumors are needed to better ascertain the additional health burden caused by the COVID-19 pandemic.

## 5. Conclusions

At our institution, during the COVID-19 pandemic, the reduced surgical activity for urologic tumors did not lead to an increase in the average waiting time from diagnosis to surgery and in emergency hospital admissions for urologic cancers. In accordance with recommendations by international urological societies, surgeries for high-risk urological tumors were prioritized during COVID-19 pandemic. However, future implications for patients’ prognosis derived from the decrease in diagnostic and therapeutic procedures for low-risk asymptomatic tumors still need to be determined.

## Figures and Tables

**Table 1:** Characteristics of patients undergoing urologic oncology surgeries in 2019 and 2020.

	2019 (*n* = 720)	2020 (*n* = 528)	*p*-Value
Sex			**0.012**
Male	619 (86%)	426 (81%)	
Female	101 (14%)	102 (19%)	
Age (years)	69 ± 10	69 ± 11	0.1396
Nationality			0.667
Italian	696 (97%)	508 (96%)	
Others	24 (3%)	20 (4%)	
Type of hospital admission			0.094
Elective	689 (96%)	494 (94%)	
Emergency	31 (4%)	34 (6%)	
Length of hospital stay (days)	4 (IQR 3–5)	3 (IQR 3–6)	0.8274
Indication for surgery			**<0.0001**
Kidney tumor	97 (13%)	89 (17%)	
UTUC (including renal pelvis)	9 (1%)	19 (3%)	
Bladder tumor	350 (49%)	295 (56%)	
Prostate tumor	264 (37%)	125 (24%)	
Type of procedure			**<0.0001**
Partial nephrectomy	75 (10%)	62 (12%)	
Radical nephrectomy/nephroureterectomy	31 (4%)	46 (9%)	
TURBT	318 (44%)	258 (49%)	
Radical cystectomy	32 (5%)	37 (7%)	
Radical prostatectomy	264 (37%)	125 (23%)	

Abbreviations: *n*, number; IQR, interquartile range; UTUC, upper tract urothelial carcinoma; TURBT, transurethral resection of bladder tumor. Bold values denote *p*-values < 0.05.

## Data Availability

The data presented in this study are available on request from the corresponding author.
